# Is Cerebrolysin Useful in Psychiatry Disorders?

**DOI:** 10.3390/biomedicines13071661

**Published:** 2025-07-08

**Authors:** Szymon Florek, Patryk Główczyński, Karina Badura-Brzoza, Robert Pudlo

**Affiliations:** 1 Department of Psychoprophylaxis, Faculty of Medical Sciences in Zabrze, Medical University of Silesia in Katowice, 42-612 Tarnowskie Góry, Poland; rpudlo@sum.edu.pl; 2 Department od Psychiatry, Faculty of Medical Sciences in Zabrze, Medical University of Silesia in Katowice, 42-612 Tarnowskie Góry, Poland; patryk.glowczynski@sum.edu.pl (P.G.); kbbrzoza@sum.edu.pl (K.B.-B.)

**Keywords:** cerebrolysin, psychiatry, schizophrenia, bipolar disorder

## Abstract

**Background/Objectives**: Cerebrolysin is a well-known mixture of peptides that has been used for many years, primarily in patients with neurological disorders. Thanks to its unique properties, this substance supports endogenous repair mechanisms and protects the brain from damaging factors. Cerebrolysin is most widely used in Eastern European countries. However, data on the potential use of cerebrolysin in mental disorders are difficult to find in the literature. This review focuses on the potential use of cerebrolysin in psychiatry, and two independent researchers searched three full-text medical article databases to compile it. **Methods**: To conduct this scoping review, two independent researchers searched three full-text article databases, Embase, Scopus, and Web of Science, by entering the following phrases: “cerebrolysin psychiatry”, “cerebrolysin depression”, “cerebrolysin mood”, “cerebrolysin bipolar”, “cerebrolysin schizophrenia”, and “cerebrolysin addiction”. **Results**: The results show that this specific substance could have a relatively small application in psychiatry. **Conclusions**: The limited amount of available research on the use of cerebrolysin suggests that it may have some significance in supporting the treatment of depression and autism spectrum disorders and alleviating adverse effects during treatment with neuroleptics.

## 1. Introduction

Cerebrolysin is a multimodal neuropeptide obtained from structural proteins in pig brain. It consists of low molecular weight peptides (25%) and free amino acids (75%) [1]. It also contains 25 other microelements, including magnesium, potassium, calcium, selenium, and zinc [2]. Cerebrolysin also owes its unique properties to its low molecular weight, which allows it to freely cross the blood–brain barrier [3]. This substance was invented in 1949 in Innsbruck, but it gained particular popularity in Eastern European countries [4,5]. Cerebrolysin is currently used in 44 countries, primarily in Europe and Asia. Interestingly, the FDA (U.S. Food and Drug Administration) has not registered this substance for use in the United States [6]. Although there is no specific data on the number of centres using cerebrolysin in everyday clinical practice, an international study conducted in 2024 included only 19 centres from 10 countries, which may reflect the low popularity of this drug even in countries where it is available [7]. The mechanism of action of the drug is complex and involves the propagation of the signaling of the Shh pathway by modulating mRNA synthesis [8]. The action of cerebrolysin could also be similar to that of the neurotrophic growth factor NTF3 [9], and the substance may also have a stimulating effect on the production of BDNF (brain-derived neurotrophic growth factor) [10]. Clinically, cerebrolysin is used primarily for neurorehabilitation in patients after stroke [5] or other injuries to the central nervous system [11]. One study showed that cerebrolysin administered in the subacute phase of ischemic stroke in combination with neurological rehabilitation was more effective than rehabilitation alone and helped improve motor function [12]. Furthermore, numerous studies have been conducted on its use as a substance to support the treatment of dementia, especially the vascular and Alzheimer’s types [13,14]. A randomized study has shown that cerebrolysin may also be used in the treatment of aphasia [15]. Some authors point out that not all mechanisms of action of cerebrolysin have been sufficiently understood, which opens up potential possibilities for the use of the drug in other diseases [16].

Probably its most popular use is supporting recovery after acute ischemic stroke. A meta-analysis conducted in 2021 indicates a very good safety profile of the substance [17]. On the other hand, a large systematic review suggests moderate certainty regarding the effectiveness of preventing death after stroke; moreover, evidence of the same strength emphasizes an increase in serious nonfatal adverse events. Such events included, among others, acute coronary syndrome, atrial fibrillation, heart failure, gastric ulcer, pneumonia, rectal cancer, coma, pleural effusion, aspiration pneumonia, cerebral hematoma, and pulmonary embolism [5]. However, a systematic review with a meta-analysis conducted in 2023 on the use of cerebrolysin among patients with subarachnoid hemorrhage suggests a possible reduction in mortality in this group of patients as a result of using the substance in question [18].

Dementia is a group of diseases of interest to both psychiatry and neurology. In the ICD-10 classification, dementia is placed in the chapter on mental disorders and, additionally, in the chapter on neurological disorders [19], while in ICD-11, it is placed in the chapter on diseases of the nervous system [20]. A broad systematic review of the use of cerebrolysin in vascular dementia proves its positive effect on cognitive performance and general functioning in people with mild or moderate dementia. However, the authors emphasize the lack of strong evidence that would justify the introduction of cerebrolysin as a routine treatment for this type of dementia [21].

Considering the mechanism of action of cerebrolysin, it seems that an attempt to use it in the above-described diseases could bring measurable benefits. It also seems that this substance could be used in some psychiatric conditions. There is increasing scientific evidence indicating the participation of brain cell degradation processes in the etiology of mental disorders such as schizophrenia or depression [22,23]. An analysis of the possibilities of using cerebrolysin in this area of medicine could bring specific benefits. However, the specific conditions of administration of the substance—only in the intravenous form—may make it somewhat difficult to select a group of patients in whom it could be used.

There are a number of studies available on the use of cerebrolysin in neurological conditions, including dementia, but there are relatively few reports on the use of this substance in mental disorders. The aim of this review is to analyze the studies on the use of cerebrolysin in psychiatry. It was extended with a bibliometric analysis of publications on cerebrolysin in order to identify regions of the world with the largest number of articles on the subject.

## 2. Materials and Methods

To conduct this scoping review, two independent researchers searched 3 full-text article databases, Embase, Scopus, and Web of Science, by entering the following phrases: “cerebrolysin psychiatry”, “cerebrolysin depression”, “cerebrolysin mood”, “cerebrolysin bipolar”, “cerebrolysin schizophrenia”, and “cerebrolysin addiction”. In each database, search terms were used with the conjunction ‘AND’ to search the entire article, without limiting the search to the title of the publication. The criterion for inclusion of articles in this review was the availability of publications in English. It should be emphasized that this was the only inclusion criterion. Articles on the use of cerebrolysin in neurological disorders or only on animals were excluded. Due to the limited number of articles covering the subject area under study, the authors decided not to restrict the publication dates or types of articles in any way.

### Bibliometric Analysis

**Figure 1:**
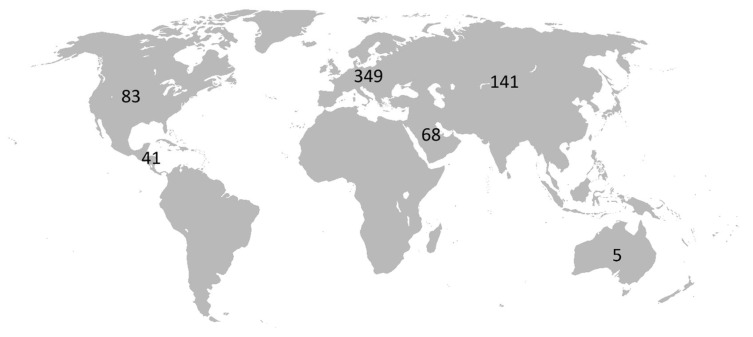
Distribution of the number of articles on cerebrolysin in the world.

Scopus retrieved 771 articles from 1995 to 2025. The largest number of articles came from Europe (349 publications), followed by Asia (including the Russian Federation, 141 publications); North America (US and Canada, 83 publications); the Arabian Peninsula (68 publications), Latin America (41 publications), and Australia and New Zealand (5 publications). The country that published the most articles on cerebrolysin was the Russian Federation (133 articles). Searches using Web of Science and Embase generated results that were similar to those of Scopus: the European country that published the most articles was Austria. The geographical distribution of publications is presented in Figure 1.

## 3. Results

**Figure 2:**
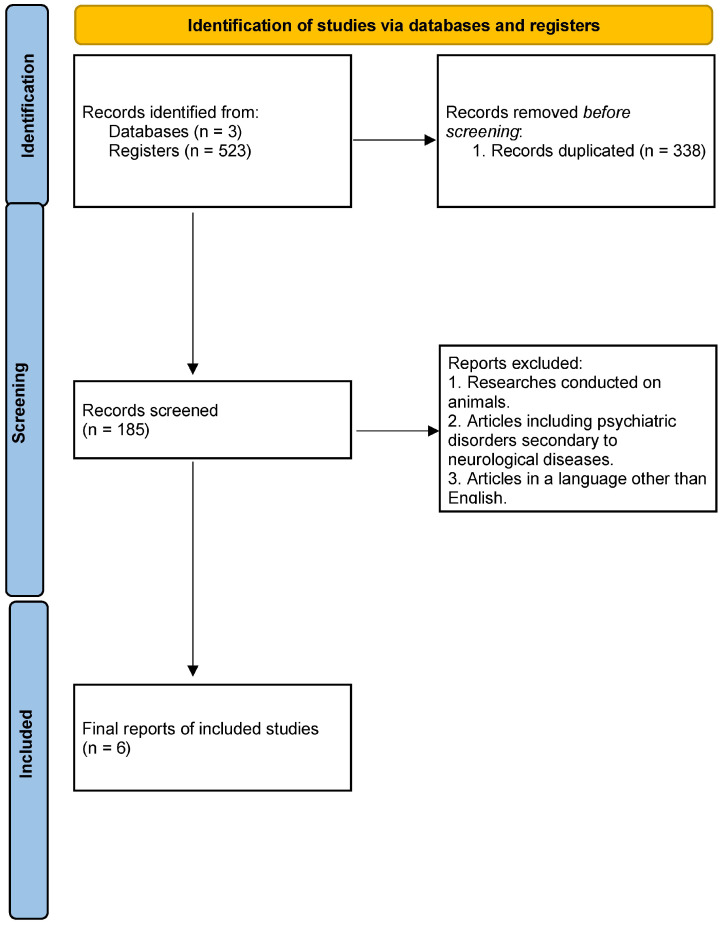
Article qualification process.

The search yielded a total of 523 articles, of which 338 were duplicates. The remaining 185 articles were subjected to a rigorous analysis, as shown in Figure 2. Finally, six articles were included in this review, of which two were research papers, one was a letter to the editor, and three were conference abstracts. The article qualification process is shown in Figure 2.

The articles analyzed in this review are summarized in Table 1.

In Table 1, it is noteworthy that there are only two original articles, and both concern psychotic disorders. In the latest study from 2023, a total of 59 patients participated, of which only 5 patients received treatment with cerebrolysin. The results emphasized that cerebrolysin, similar to citicoline, cortexin, actovegin, gliatilin, and 2-ethyl-6-methyl-3-hydroxypyridine succinate (EMHS), has a protective effect on the side effects of neuroleptics [24]. In a slightly older Chinese–Australian study, 109 patients diagnosed with schizophrenia were recruited. This is a randomized study—55 people were given cerebrolysin together with risperidone, and the remaining 54 were given placebo instead of cerebrolysin, and intravenous therapy in both cases lasted 4 weeks, from Monday to Friday. This study demonstrated the potential of cerebrolysin to improve cognitive functions in patients with predominantly negative symptoms related to schizophrenia [25]. In the letter to the editor, the author reports on the efficacy of cerebrolysin in autism and Alzheimer’s disease [26].

The remaining items included in the review are post-conference abstracts. Pochueva et al. conducted a study covering 41 people diagnosed with a mild or moderate depressive episode. The respondents were divided into two groups, one of which received actovegin and the other cerebrolysin; the study did not include a control group. The results indicated that both methods of augmenting antidepressant treatment may be beneficial [27]. In the Ukrainian study, 54 adolescent girls aged 12–14 years were examined, and cerebrolysin was also among the treatment methods used. The conclusions refer only to the need for a special approach to people with anorexia [28]. In the last publication included in the review, six children aged 3–8 years suffering from developmental disorders were examined and given cerebrolysin, which resulted in positive effects [29].

## 4. Discussion

The mechanism of action of cerebrolysin (which could potentially be the focus of interest if we take into account psychiatric diseases) involves the stimulation of BDNF synthesis. This is a protein that determines neuroplasticity through both neurogenesis and neuroregeneration. Scientific studies show that SSRIs (serotonin reuptake inhibitors) increase the concentration of BDNF, and initially, low BDNF concentrations are seen as the genesis of affective disorders. On the other hand, lithium carbonate treatment, depending on the brain region, has an inhibitory or stimulating effect on the BDNF level [30]. It is worth noting that electroconvulsive therapy can also lead to an increase in BDNF concentration [31]. Assuming a certain importance of BDNF in the etiology of depression and the fact that changes in its concentration may be related to the occurrence of affective disorders, it would be worth trying to conduct research to assess the potentiating effect of cerebrolysin in the treatment of the above diseases. Additionally, the genesis of depressive disorders [32] is also believed to be caused by low concentrations of the third neurotrophin (NTF3), and cerebrolysin can imitate its action.

Based on our review of the literature, it can be concluded that the available data on the use of cerebrolysin in mental disorders are limited. This applies to both the number of studies and their methodological quality. The results of the analyzed studies suggest promising, albeit preliminary, findings. However, this fact seems surprising in light of the possible effects of cerebrolysin in mental disorders presented in the introduction and its use over many decades. Two original studies conducted on patients diagnosed with schizophrenia indicated a beneficial effect of cerebrolysin on cognitive symptoms and the alleviation of adverse effects of antipsychotic drugs [24,25]. Other publications, including post-conference reports, suggest the possibility of using cerebrolysin in the treatment of depression, anorexia, and developmental disorders and as an adjunct in autism and Alzheimer’s disease, but the lack of control groups and the limited number of participants significantly weaken the strength of these conclusions [26,27,28,29].

It seems intuitive to conduct research on the effect of cerebrolysin on mood disorders among geriatric patients because in this group of patients, depressive disorders may be prodromal for dementia, according to Pochueva et al. The addition of cerebrolysin to antidepressant treatment proved to be an effective pharmacological intervention, recommended by the authors for use in hospital wards [27]. The supportive role of cerebrolysin was demonstrated to improve cognitive functions in adolescent patients with eating disorders accompanied by depressive disorders [28]. Some authors indicate that cerebrolysin may have a potentially beneficial effect on avolition in the course of depression [33]. Referring to the above-mentioned mechanisms of action of cerebrolysin, its effects on depressive disorders should come as no surprise, and should serve as a starting point for further exploration of these areas of science.

Furthermore, a positive effect of cerebrolysin in alleviating side effects such as dysuria or extrapyramidal symptoms appearing during treatment with neuroleptics in a group of patients diagnosed with late-onset schizophrenia was documented [24]. Another study presented an attempt to alleviate cognitive disorders with cerebrolysin among patients taking neuroleptics, not only in the course of schizophrenia but also in the context of autism spectrum disorder with accompanying behavioral disorders [29]. In the study by Xiao et al., adding cerebrolysin to risperidone for the purpose of augmenting the treatment of psychotic symptoms did not increase the efficacy of risperidone, but a positive effect on improving cognitive functions in the study group was confirmed [25]. Observations concerning the addition of cerebrolysin to neuroleptics are undoubtedly interesting and emphasize extremely important elements of treatment related to adverse effects and cognitive functions. Unfortunately, given the need for intravenous administration of cerebrolysin, augmentation of neuroleptic treatment with this substance is unlikely to become widely used.

In available medical databases, one can find a study by Hosseini et al. on the effect of cerebrolysin on the symptoms of post-traumatic stress disorder and cognitive functions in mice (measured by the concentration of steroid hormones and neurotransmitters, i.e., synaptophysin). This study showed that cerebrolysin had a beneficial effect by reducing anxiety and improving cognitive functions [34]. Unfortunately, there are no similar studies in humans.

In summary, despite the potential efficacy of cerebrolysin in mental disorders, there are very few reports on this subject in the literature. This may be due to two main reasons. Firstly, the currently known and used treatments for mental disorders are relatively effective. In depressive disorders, oral treatment with SSRIs, SNRIs, and other drugs is effective. In drug-resistant depression, electroconvulsive therapy, intranasal esketamine, or intravenous ketamine are appropriate [35]. In light of the data collected, it can be concluded that these methods are more effective than cerebrolysin. The situation is similar in schizophrenia, where clozapine or electroconvulsive therapy is used in cases of drug resistance [36]. Based on the collected research, it can be suspected that cerebrolysin may help alleviate the side effects of the drugs used. However, although this field seems interesting, the limitations resulting from the second issue, i.e., the intravenous form of cerebrolysin administration, significantly limit its wider use. It should be noted here that intravenous administration is much less comfortable, stigmatising, and requires frequent visits to the clinic, which discourages patients from continuing treatment. Furthermore, according to the drug leaflet, the infusion must be diluted to a volume of 100–250 mL and administered daily over 15–60 min for at least 10–20 days [37]. As a result, despite the promising effects of cerebrolysin, its intravenous form of administration limits the practical implementation and scalability of the therapy in outpatient psychiatry.

To the best of the authors’ knowledge, this article is the first Perspective-type study devoted to the analysis of the potential applications of cerebrolysin in the treatment of mental disorders. Unlike a systematic review, this type of publication is not based on strictly defined criteria for the selection and evaluation of research quality, which may limit the generalisability of the conclusions presented. Despite an extensive search of databases with no age restrictions, only six publications meeting the above inclusion criteria were ultimately identified. Only two of these studies are full-text original studies, while the rest are conference abstracts or letters to the editor, which significantly reduces the scientific value of the review. The authors of the review focused exclusively on studies conducted in humans, foregoing the analysis of animal model experiments, which were few in number and were briefly discussed in the Section 4.

## 5. Conclusions

1. The small number of studies available on the use of cerebrolysin indicates that it may have some significance in alleviating adverse effects and improving cognitive functions during treatment with neuroleptics.
2. There are isolated reports concerning the potential efficacy of cerebrolysin in augmenting the treatment of depression, anorexia, autism spectrum disorder, and pervasive developmental disorders.
3. In the future, it would be beneficial to design and conduct studies with a particular emphasis on the use of cerebrolysin in supporting the treatment of mental disorders.

## Figures and Tables

**Table 1:** Summary of articles included in the review.

Author	Country, Year of Publication	Study Design	Simple Description	Intervention	Conclusions
Boksha et al. [15]	Russia, 2023	Original article, experimental, clinical trial	Late-onset psychosis, 59 patients (56 females, 3 males), 5 people with cerebrolysin	2152 mg per day for 10 days	Cerebrolysin reduces the side effects of neuroleptics.
Kapoor [16]	USA, 2012	Letter to the editor, opinion	Autism spectrum disorder	-	Cerebrolysin, when administered in combination with neuroleptics, may alleviate cognitive impairment in patients with mild to moderate autism. It may also alleviate side effects.
Xiao et al. [17]	China, 2012	Original article, experimental, placebo-controlled	Schizophrenia, 52 people with cerebrolysin (41 male, 11 female), 49 with placebo (34 male, 15 female)	30 mL of cerebrolysin in 250 mL physiological saline intravenous infusion; the placebo group: 30 mL of placebo in 250 mL physiological saline intravenous infusion daily from Monday to Friday over 4 weeks.	Cerebrolysin did not increase the efficacy of risperidone in treating positive and negative symptoms, but improved cognitive function.
Pochueva et al. [18]	Russia, 2024	Conference, experimental, clinical trial	Depression, 21 people (7 men, 14 women) with actovegin; 20 patients (5 men, 15 women) with cerebrolysin	No data available	Cerebrolysin is effective in treating depression in a geriatric population.
Mykhailova et al. [19]	Ukraine, 2021	Conference, experimental, clinical trial	Anorexia nervosa, 54 adolescent girls with cerebrolysin	Cerebrolysin 10.0 with 0.9 % sodium chloride 200.0, no more data available	Depression in anorexia requires a special approach.
Mosawi et al. [20]	Iraq, 2019	Conference, experimental, clinical trial	Pervasive developmental disorders, 6 patients with cerebrolysin	Courses of cerebrolysin were given in individualized	Significant reduction in autistic features, with some patients showing complete disappearance of core autistic features.

## Data Availability

No new data were created.
